# Supplementary material for: The cost-effectiveness of controlling dengue in Indonesia using wMel Wolbachia released at scale: a modelling study
Source: BMC Med. 2020 Jul 9;18:186. doi: 10.1186/s12916-020-01638-2 (PMC7346418; doi:10.1186/s12916-020-01638-2)
Supplement: Supplementary file 1 — Additional file 1. Additional information on the methods and results of the main manuscript. Includes further details on the timing of different phases in different scenarios (S1.1), cost of release data (S1.2), more detailed methods on the cost of release model and its fit (S1.3), the parameter values and rational for long-term cost reductions (S1.4), further background on specific challenges to the establishing Wolbachia and how they can be overcome (S1.5) and maps of cost effectiveness with more detailed Ordinance survey base maps (S1.6). [file 12916_2020_1638_MOESM1_ESM.docx]

# Supplementary Information for: “The cost-effectiveness of controlling dengue in Indonesia using *wMel* *Wolbachia* released at scale: a modelling study”

# S1.1 Timelines of programme phases and benefits acquisition

| Programme year | Accelerated | Sequenced | Programme challenges | | | | | | |
| --- | --- | --- | --- | --- | --- | --- | --- | --- | --- |
|  |  |  | Low coverage | Low coverage fixed | Initially uncompetitive with natural mosquito population | Emergence of resistance | Emergence of resistance fixed | Reliance of passive disease surveillance | Innovation efficiencies and economies of scale |
| 1 | Phase 1 | Phase 1 | Phase 1 | Phase 1 | Phase 1 | Phase 1 | Phase 1 | Phase 1 | Phase 1 * 50% |
| 2 | Phase 1 | Phase 1 | Phase 1 | Phase 1 | Phase 1 | Phase 1 | Phase 1 | Phase 1 | Phase 1 * 50% |
| 3 | Phase 2 | Phase 2 | Phase 2 | Phase 2 | Phase 2 | Phase 2 | Phase 2 | Phase 2 | Phase 2 * 50% |
| 4 | Phase 3 | Phase 2 | Phase 3 | Phase 3 | Phase 1 | Phase 3 | Phase 3 | Phase 3 | Phase 3 * 50% |
| 5 | Phase 3 | Phase 2 | Phase 3 | Phase 2 | Phase 2 | Phase 3 | Phase 3 | Phase 3 | Phase 3 * 50% |
| 6 | Phase 3 | Phase 2 | Phase 3 | Phase 3 | Phase 3 | Phase 3 | Phase 3 | Phase 3 | Phase 3 * 50% |
| 7 | Phase 4 | Phase 2 | Phase 4 | Phase 3 | Phase 3 | Phase 4 | Phase 4 |  | Phase 4 * 50% |
| 8 | Phase 4 | Phase 2 | Phase 4 | Phase 3 | Phase 3 | Phase 4 | Phase 4 |  | Phase 4 * 50% |
| 9 | Phase 4 | Phase 2 | Phase 4 | Phase 4 | Phase 4 | Phase 4 | Phase 1 |  | Phase 4 * 50% |
| 10 | Phase 4 | Phase 2 | Phase 4 | Phase 4 | Phase 4 | Phase 4 | Phase 2 |  | Phase 4 * 50% |
| 11 | Phase 4 | Phase 3 | Phase 4 | Phase 4 | Phase 4 | Phase 4 | Phase 3 |  | Phase 4 * 50% |
| 12 | Phase 4 | Phase 3 | Phase 4 | Phase 4 | Phase 4 | Phase 4 | Phase 3 |  | Phase 4 * 50% |
| 13 | Phase 4 | Phase 3 | Phase 4 | Phase 4 | Phase 4 | Phase 4 | Phase 3 |  | Phase 4 * 50% |
| 14 |  | Phase 4 |  | Phase 4 | Phase 4 |  | Phase 4 |  |  |
| 15 |  | Phase 4 |  | Phase 4 | Phase 4 |  | Phase 4 |  |  |
| 16 |  | Phase 4 |  |  |  |  | Phase 4 |  |  |
| 17 |  | Phase 4 |  |  |  |  | Phase 4 |  |  |
| 18 |  | Phase 4 |  |  |  |  | Phase 4 |  |  |
| 19 |  | Phase 4 |  |  |  |  | Phase 4 |  |  |
| 20 |  | Phase 4 |  |  |  |  | Phase 4 |  |  |

Dark green shading indicates years in which benefits accrue. Light green shading indicates years in which benefits accrue at a reduced rate (due to 50% intervention coverage).

# S1.2 Cost of release data

*Budgeted cost per km^2^ of Wolbachia release programmes in various countries*

| Location | Programme Phase | Human population density (people per km^2^) | Type of release | National GDP (PPP) | Cost per km^2^ release area (USD) | Population | Area covered (km^2^) |
| --- | --- | --- | --- | --- | --- | --- | --- |
| Yogyakarta, Indonesia | 2 | 11,859 | Eggs | 12,378 | 60,068 | 225,321 | 19 |
| Medellin, Colombia** | 1 | 10,043 | Adults | 14,455 | 194,362 | 188,199 | 18.74 |
| Medellin, Colombia** | 2 | 11,440 | Adults | 14,455 | 41,800 | 729,739 | 63.79 |
| Medellin, Colombia** | 2 | 11,832 | Adults | 14,455 | 42,202 | 815,111 | 68.89 |
| Colombo, Sri Lanka* | 1 | 25,268 | Eggs | 13,001 | 124,298 | 78,331 | 2.28 |
| Colombo, Sri Lanka* | 2 | 6,503 | Eggs | 13,001 | 58,039 | 83,824 | 9.96 |
| Colombo, Sri Lanka* | 2 | 9,435 | Eggs | 13,001 | 58,503 | 114,352 | 9.44 |
| Townsville, Australia | 1 | 2,425 | Eggs | 49,882 | 54,175 | 49,224 | 20.3 |
| Townsville, Australia | 2 | 2,311 | Eggs | 49,882 | 27,115 | 42,068 | 18.2 |
| Townsville, Australia | 2 | 1,232 | Adults | 49,882 | 17,531 | 21,687 | 17.6 |
| Townsville, Australia | 2 | 2,761 | Adults | 49,882 | 27,963 | 26,778 | 9.7 |
| Vanuatu | 1 | 1,333 | Eggs | 2,780 | 18,831 | 44,000 | 33 |

Notes: GDP = gross domestic product; PPP = purchasing power parity

* For Sri Lanka (Colombo) field release costs (excluding preparation and planning) were not explicitly disaggregated by area, so we assumed field release costs were proportional to the total number of *Wolbachia* mosquitoes released in each area.

** For Colombia (Medellin and Bello), Budgetary Phases 1 and 2B.1 were merged to give a programmatic Phase 1 scale that was consistent with our definition of a Phase 1 programme and more comparable to the aims of Phase 1 programmes in Sri Lanka and Australia. WMP consultancy, equipment, travel and personnel costs were also redistributed from budgetary Phase 1 equally across all Phases. Finally, budgetary Phase 1 for Colombia includes establishing and fitting out a new insectary with capacity to produce mosquitoes for the whole of Antioquia Department which costs significantly more than would be required for just the release areas in Phase 1 and 2 of the Medellin and Bello programme. Therefore, we decided to apportion the cost of this new facility proportional to the fraction of the population of Antioquia who lived in the release area (1.7 million / 6.3 million = 0.270).

# S1.3 Model formula for cost of release model

$$\log\left( C_{A} \right) \sim\log(\alpha P_{A})+\beta S_{p}+\gamma E_{p}+\delta G_{A}+ \tau$$

Where:

$C_{A}$ = Cost per km^2^ release area

$P_{A}$ = Human population density (people per km^2^)

$S_{p}$ = Programme phase

$E_{p}$ = Release material (eggs or adults)

$G_{A}$ = National GDP

$\alpha, \beta, \gamma, \delta, \tau$ = coefficients and intercepts fit by the model

Model was fit using lease squares using the glm() base function in R version 3.3.3.

The final fitted model coefficients were as follows:

| Variable | Mean coefficient | p-value (two sided t-test) |
| --- | --- | --- |
| Intercept | 2.6500 | 0.00391 |
| Human population density in release area | 0.6499 | 0.00323 |
| Programme phase | -0.3009 | 0.02633 |
| Release material = eggs (among eggs or adults) | -0.002355 | 0.98227 |
| National GDP | 0.000003858 | 0.30666 |

Fit comparison between models with a response variable of cost per km^2^ and cost per person

| Model | Pearson’s correlation coefficient |
| --- | --- |
| Cost per km^2^ | 0.8123707 |
| Cost per person | 0.7445477 |

# S1.4 Long term costs of *Wolbachia* deployment

| **Cost category** | **Annual cost in Phase 3 (as % of Phase 2 annual cost)** | **Annual cost in Phase 4 (as % of Phase 2 annual cost)** | **Justifications** |
| --- | --- | --- | --- |
| Mosquito rearing | 10% | 5% | Only needed to fill in gaps (assumed conservatively to occur in 20% of release area). In 3 years experience, gaps have occurred occasionally, i.e. 1 of 12 intervention clusters in existing trial; in part due to insecticide risk |
| Release | 10% | 5% | Only needed to fill in gaps (assumed conservatively to occur in 20% of release area). In 3 years experience, gaps have occurred occasionally, i.e. 1 of 12 intervention clusters in existing trial; in part due to insecticide risk |
| Monitoring | 8% | 2% | Phase 3: 8% = 1/13, cut from weekly to quarterly monitoring – once 60% of trapped mosquitos have Wolbachia, reduce frequency of monitoring; Ph4: 2% as cut to annual monitoring of mosquitoes on rotating basis around city |
| Laboratory | 25% | 2% | Ph 3: Lab tests dengue cases in humans from community to see whether they are dengue (100%); checking mosquitoes to look for Wolbachia (8%). The routine surveillance system monitors only DHF. The World Mosquito Program would supplement with NS1 testing; Then confirm with PCR for quality checking. Fewer cases need confirmatory testing. Confirm that at least 60% of mosquitoes have Wolbachia |
| Diagnostic | 25% | 0% | Testing of patients with suspected dengue with NS1 and IgG IgM (dengue duo); |
| Communications | 10% | 5% | Population aware, minimal ongoing engagement to gap filling releases. Same as gaps (includes mass media, leaflets, posters) |
| Community engagement | 10% | 5% | Ensure that community accepts mosquito release in their area (one-on-one communications) |
| Data management | 8% | 2% | Cut from weekly to quarterly. Surveillance data; summarize and provide feedback. Might be folded into MOH reports |
| Administration and management | 20% | 5% | Cut by half proportional to ongoing need |
| Surveillance | 50% | 5% | Monitoring human infections; set up puskesmas as surveillance site; Slight reduction proportional to reduced data from surveillance, but infrastructure costs such as online data platforms remain. WMP does advocacy and helps provide rapid tests for the entomology system; since 2017 has been measuring dengue fever, not just DHF. |
| Policy Advocacy | 25% | 5% | Emphasizing need for surveillance system. Support for high level stakeholders. Policy briefs for national stakeholders providing updates on study After deployment transfer responsibility for updates to routine activities of the City Health Department, only ¼ as many by Wolbachia program. |
| WMP Global | 25% | 5% | Cut by three quarters proportional to ongoing need similar to policy advocacy |
| Overall* |  |  | - |

* Overall figure includes unequal weighting of the above categories in the budget.

# S1.5 Sensitivity of cost effectiveness to specific challenges

## Low coverage

Due to heterogeneity in local ecology of *Ae. aegypti* populations or local community objections, it may not be possible to reach 100% coverage of *Wolbachia* in the target release area (“low coverage” scenario). We therefore assess cost effectiveness if only 50% coverage with *Wolbachia* (baseline 100% coverage) can be reached using effectiveness models from a previous analysis [19]. We also test a formulation of the programme where these gaps in coverage can be subsequently filled in after. This “overcoming low coverage” scenario results in an additional Phase 2 (release) year to take place after low coverage is detected in the first Phase 3 (post release monitoring) year.

Initially uncompetitive with the natural population. For *Wolbachia* to spread efficiently through the natural *Ae. aegypti* population at deployment, the lab-reared mosquitoes need to have a similar or better fitness than their wild-type counterparts. One major reason this may not occur is if local insecticide resistance profiles differ, leaving *Wolbachia*-infected mosquitoes at increased susceptibility to local control efforts [13]. This would manifest in a failure to establish *Wolbachia* in the target population and would require further backcrossing with wild type *Ae. aegypti* and repeating releases. We model this as an additional Phase 1 and Phase 2 deployment in the target area, delaying the acquisition of benefits by two years.

## Emergence of resistance

There are three main potential concerns about the natural emergence of “resistance” to this intervention, where changes in the *Ae. aegypti* mosquito, *Wolbachia* bacterium or the locally circulating DENV themselves result in a collapse in effectiveness. Despite being almost ubiquitous among insects, no *Wolbachia* strain has been shown to naturally infect *Ae. aegypti*. This fact, combined with life history studies demonstrating fitness costs associated with *Wolbachia* infection [26], point to the biological pressure in the mosquito population for selecting *Wolbachia* resistance development. This pressure may manifest initially as a reduction in proliferation of the endosymbiont within the *Wolbachia*-infected mosquitoes. This, in turn, would impact both the viral-blocking ability as well as the vertical transmission of *Wolbachia* to the next generation and result in loss of *Wolbachia* from the target population. Another manifestation might arise if the viral-blocking component of *Wolbachia* itself has a net-positive fitness cost [27]. In that case, it would be expected that a mutant *Wolbachia* strain minus the viral-blocking component would have a competitive edge and spread faster than the original [28]. Finally, recent studies have indicated that the virus-blocking efficacy of *Wolbachia* is virus-titre dependent [29]. Sustained exposure to *Wolbachia* would thereby be expected to select for more virulent dengue infections (i.e., with higher blood titres). This would simultaneously reduce further the viral-blocking efficacy of *Wolbachia* and increase the symptomatic rate of disease. Removal of these ineffective, deviant or potentially harmful Wolbachia strains could be achieved with the release of an additional strain superinfected with multiple *Wolbachia* strains [30]. For the purpose of this analysis we assume that resistance doesn’t emerge until 5 years post release (2^nd^ year of Phase 4) and that the effects can be reversed in an “overcoming resistance” scenario in which new Phase 1 and Phase 2 years prepare and release mosquitoes infected with a new *Wolbachia* strain.

Reliance on passive surveillance

As an early stage intervention and with existing programmes having a high focus on research activities, there is the expectation that some costs may decrease as *Wolbachia* proves its reliability over time. One area where this is particularly apparent is in long-term monitoring (Phase 4) where entomological surveillance could be discontinued entirely and any signs of *Wolbachia* failure detected through the detection of dengue cases at official healthcare facilities. We model this by eliminating Phase 4 costs.

## Innovation, efficiencies and economies of scale

Existing Wolbachia release programmes, to date, have included substantial investments in research activities (e.g. additional mosquito trapping in boundary regions and additional data collection to establish pre-trial baseline conditions) that would likely not be present in operational releases once the effectiveness of the intervention has been proven in multiple settings, thus improving cost efficiency. New innovations are also being piloted including releases using unmanned aerial vehicle technology [31] that offer the potential for considerably reduced deployment costs, particularly when deployed at scale. Finally, the cost of many centralised aspects of the programme such as the costs of rearing equipment, mosquito traps, laboratory equipment for testing for Wolbachia and the Wolbachia infected mosquitoes themselves are expected to reduce in cost substantially when mass produced at scale for use across many large sites. While it is outside the scope of the current study to estimate the exact cost percentage reduction of each of these innovations, efficiencies and economies of scale, we assume a simple 50% cost reduction across all phases of the programme to estimate the potential gains in cost effectiveness of Wolbachia that could be achieved through further cost reduction strategies.

# S1.6 Maps of cost effectiveness with Ordinance survey base maps

*Figure S1: Maps of the gross cost-effectiveness of accelerated Wolbachia releases in Yogyakarta City (A), Yogyakarta SAR (B), Jakarta (C) and Bali (D). Cost effectiveness is measured in present value 2018 USD per Disability Adjusted Life Year (DALY) averted with green areas being most favourable and red areas being least favourable. These maps are analogous with figure 3 of the main manuscript but display ordinance survey base maps for local reference.*
