# Supplementary material for: The cost-effectiveness of controlling dengue in Indonesia using wMel Wolbachia released at scale: a modelling study
Source: BMC Med. 2020 Jul 9;18:186. doi: 10.1186/s12916-020-01638-2 (PMC7346418; doi:10.1186/s12916-020-01638-2)
Supplement: Supplementary file 2 — Additional file 2. The ISPOR Consolidated Health Economic Evaluation Reporting Standards (CHEERS) checklist. [file 12916_2020_1638_MOESM2_ESM.docx]

| Section/item | Item No | Recommendation | Reported on Page no. / line no. |
| --- | --- | --- | --- |
| Title | 1 | Identify the study as an economic evaluation or use more specific terms such as “cost-effectiveness analysis”, and describe the interventions compared. | Page 1, title |
| Abstract | 2 | Provide a structured summary of objectives, perspective, setting, methods (including study design and inputs), results (including base case and uncertainty analyses), and conclusions. | Page 2, throughout Abstract |
| Background and Objectives | 3 | Provide an explicit statement of the broader context for the study. Present the study question and its relevance for health policy or practice decisions. | Broad context in firth three paragraphs of the introduction, specific questions addressed and relevance in an Indonesian setting in paragraphs 4 and 6 |
| Target groups and sub-populations | 4 | Describe characteristics of the base case population and subgroups analysed, including why they were chosen. | Detailed in the “Candidate release sites” subsection of the methods (page 7 line 175) |
| Setting and location | 5 | State relevant aspects of the system(s) in which the decision(s) need(s) to be made. | See the “costing of Wolbachia releases” and “costing long-term surveillance” sections” on lines 147 and 164 respectively |
| Study perspective | 6 | Describe the perspective of the study and relate this to the costs being evaluated. | See “time horizon and acquisition of benefits and discounting section” third paragraph (line 205) |
| Comparators | 7 | Describe the interventions or strategies being compared and state why they were chosen. | See “time horizon and acquisition of benefits and discounting section” third paragraph (line 210) |
| Time Horizon | 8 | State the time horizon(s) over which costs and consequences are being evaluated and say why appropriate. | See “time horizon and acquisition of benefits and discounting section” first paragraph (line 195) |
| Discount rate | 9 | Report the choice of discount rate(s) used for costs and outcomes and say why appropriate. | See “time horizon and acquisition of benefits and discounting section” second paragraph (line 200) |
| Choice of health outcomes | 10 | Describe what outcomes were used as the measure(s) of benefit in the evaluation and their relevance for the type of analysis performed. | See “Cost of dengue illness averted” section (line 169) |
| Measurement of effectiveness | 11a | Single study-based estimates: Describe fully the design features of the single effectiveness study and why the single study was a sufficient source of clinical effectiveness data. | See paragraph titled “Modelling effectiveness and cost of dengue illness averted” in the methods section |
| Measurement and valuation of preference based outcomes | 12 | If applicable, describe the population and methods used to elicit preferences for outcomes. | Not applicable |
| Measurement and valuation of preference based outcomes Estimating resources and costs | 13b | Model-based economic evaluation: Describe approaches and data sources used to estimate resource use associated with model health states. Describe primary or secondary research methods for valuing each resource item in terms of its unit cost. Describe any adjustments made to approximate to opportunity costs. | See “costing Wolbachia releases (Phases 1 and 2)” and ”Costing long-term surveillance (Phases 3 and 4)” sections (lines 147 and 164 respectively). Also see Additional file 1, S1.4. |
| Currency, price date, and conversion | 14 | Report the dates of the estimated resource quantities and unit costs. Describe methods for adjusting estimated unit costs to the year of reported costs if necessary. Describe methods for converting costs into a common currency base and the exchange rate. | See “time horizon and acquisition of benefits and discounting section” second paragraph (line 200) and Additional file 1 S1.3. |
| Choice of model structure | 15 | Describe and give reasons for the specific type of decision- analytical model used. Providing a figure to show model structure is strongly recommended. | See “Phases of the programme” section (line 124) and figure 2 |
| Assumptions | 16 | Describe all structural or other assumptions underpinning the decision-analytical model. | See “time horizon and acquisition of benefits and discounting section” all paragraphs + various sections of the results and discussion section |
| Analytical methods | 17 | Describe all analytical methods supporting the evaluation. This could include methods for dealing with skewed, missing, or censored data; extrapolation methods; methods for pooling data; approaches to validate or make adjustments (such as half cycle corrections) to a model; and methods for handling population heterogeneity and uncertainty. | See appendex 1 for detailed methods on combining cost data from multiple cites. And O”Reilly et al. BMC Medicine 2019 for analytic predictions of effectiveness. |
| Study parameters | 18 | Report the values, ranges, references, and, if used, probability distributions for all parameters. Report reasons or sources for distributions used to represent uncertainty where appropriate. Providing a table to show the input values is strongly recommended. | See tables 1 and 2 and figure 1. Input values are variable and scenario-specific so do not lend themselves to a simple input value table. |
| Incremental costs and outcomes | 19 | For each intervention, report mean values for the main categories of estimated costs and outcomes of interest, as well as mean differences between the comparator groups. If applicable, report incremental cost-effectiveness ratios. | See second paragraph of the “Cost-effectiveness” section of the results (line 301), table 3 and figure 4A. |
| Characterising uncertainty | 20b | Model-based economic evaluation: Describe the effects on the results of uncertainty for all input parameters, and uncertainty related to the structure of the model and assumptions. | See “sensitivity and uncertainty” section and figures 4B and C |
| Characterising heterogeneity | 21 | If applicable, report differences in costs, outcomes, or cost- effectiveness that can be explained by variations between subgroups of patients with different baseline characteristics or other observed variability in effects that are not reducible by more information. | See first paragraph of “cost-effectiveness” section (line 285) and figure 3 |
| Study findings, limitations, generalisability, and current knowledge | 22 | Summarise key study findings and describe how they support the conclusions reached. Discuss limitations and the generalisability of the findings and how the findings fit with current knowledge. | See paragraphs 1 (lien 328) an 7 (line 389) of the discussion respectively |
| Source of funding | 23 | Describe how the study was funded and the role of the funder in the identification, design, conduct, and reporting of the analysis. Describe other non-monetary sources of support. | See “Funding” section |
| Conflicts of interest | 24 | Describe any potential for conflict of interest of study contributors in accordance with journal policy. In the absence of a journal policy, we recommend authors comply with International Committee of Medical Journal Editors recommendations. | See “Competing interests” section. |
